# Survival of recombinant monoclonal and naturally-occurring human milk immunoglobulins A and G specific to respiratory syncytial virus F protein across simulated human infant gastrointestinal digestion

**DOI:** 10.1016/j.jff.2020.104115

**Published:** 2020-10

**Authors:** Jiraporn Lueangsakulthai, Baidya Nath P. Sah, Brian P. Scottoline, David C. Dallas

**Affiliations:** a Nutrition Program, School of Biological and Population Health Sciences, College of Public Health and Human Sciences, Oregon State University, Corvallis, OR 97331, United States; b Department of Pediatrics, Oregon Health and Science University, Portland, OR 97239, United States

**Keywords:** Palivizumab, Immunoglobulins, Monoclonal antibody, Infants, Respiratory syncytial virus, *In vitro* gastrointestinal digestion, RSV, Respiratory syncytial virus, Ig, Immunoglobulins, LLOQ, Lower limit of quantification, ULOQ, Upper limit of quantification, CV, Coefficient of variation, SD, Standard deviation, HM, Human milk, G, Gastric, I, Intestinal

## Abstract

To help rationally design an antibody for oral administration, we examined how different isotypes (IgG, IgA and sIgA) with the same variable sequence affect antibody stability across digestion. We compared the degradation of recombinant palivizumab (IgG1), and recombinant IgA and sIgA versions of palivizumab spiked in human milk to the degradation of naturally-occurring anti-respiratory syncytial virus (RSV) sIgA/IgA and IgG in human milk from four donors across gastric and intestinal phases of an *in vitro* model of infant digestion via a validated RSV F protein ELISA. Palivizumab IgG and IgA formats were less stable than the sIgA version after complete simulated gastrointestinal digestion: palivizumab IgG, IgA and sIgA decreased across complete simulated gastrointestinal digestion by 55%, 48% and 28%, respectively. Naturally-occurring RSV F protein-specific IgG was stable across digestion, whereas naturally-occurring sIgA/IgA was stable in the gastric phase but decreased 33% in the intestinal phase of simulated digestion.

## Highlights

• Naturally-occurring antibodies were more resistant to degradation than monoclonal antibodies.
• Monoclonal sIgA was more resistant to degradation than IgG and IgA.
• Monoclonal antibodies may need to be provided at a higher dose to compensate for digestive losses.

## Graphical abstract

**Figure:**
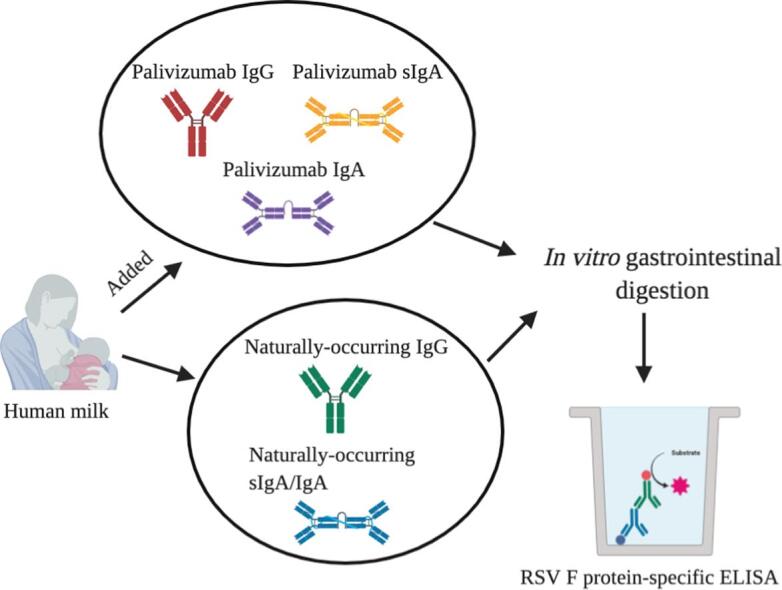

## 1 Introduction

Diarrhea is a leading cause of illness and death among children aged < 5 years in developing countries (Kotloff et al., 2012). Providing oral enteric pathogen-specific antibody supplements is a potential approach to reduce disease incidence. To prevent infection, supplemental antibodies would ideally need to resist degradation in the infant gastrointestinal tract. Palivizumab, a humanized recombinant monoclonal antibody (IgG1) that targets the human RSV prefusion F protein, is the only pathogen-specific monoclonal antibody that is Food and Drug Administration (FDA)-approved for preventing infections in high-risk infants albeit via intramuscular injection (Robbie, Zhao, Mondick, Losonsky, & Roskos, 2012). We selected palivizumab as a model for examining recombinant monoclonal antibody digestion across the gastrointestinal tract. Palivizumab was used as an available IgG as a model for digestion in infants with the goal of later translation to diarrheal pathogen-specific antibodies. This FDA approval enabled us to gain ethical approval to feed this antibody to infants in a follow-on study to this one. To help design a supplemental antibody, we examined the digestive stability of different isotypes (IgG, IgA and sIgA) of the same monoclonal antibody (palivizumab). As milk also contains naturally-occurring RSV-specific antibodies of the same classes (Mazur et al., 2019), we compared the digestion stabilities of recombinant and naturally-occurring antibodies.

By 3 years of age, nearly all children have been infected with RSV, which induces a lower respiratory tract infection (Gentile et al., 2019, Munoz et al., 2003). In infants born prematurely (< 37 weeks gestational age at birth) and infants with significant lung or heart disease, RSV infection can be severe or life-threatening (Olchanski et al., 2018). The infant immune system is underdeveloped and mostly relies on innate immunity and the presence of maternal transplacentally-transferred immunoglobulin G (IgG) or human milk-derived antibodies to protect against infectious diseases (Sparrow, Friede, Sheikh, & Torvaldsen, 2017). Ninety-seven percent of infants have RSV-specific maternal antibodies in their blood at birth (Ochola et al., 2009). Infants with high transplacental RSV-neutralizing antibody concentrations were less susceptible to RSV infection (Stensballe et al., 2009). Human milk contains RSV-specific IgG, IgA and sIgA that can neutralize RSV (Mazur et al., 2019). Though a high human milk RSV-specific IgG is correlated with lower RSV infection (Mazur et al., 2019), whether human milk antibodies provide additional protection against RSV infections beyond maternal transplacentally-transferred IgG remains unknown. Our previous study (Lueangsakulthai, Sah, Scottoline, & Dallas, 2020) revealed that naturally-occurring antibodies IgG and sIgA/IgA were more stable across *ex vivo* infant gastrointestinal digestion than monoclonal antibodies (IgG, IgA, sIgA formats). However, no study has demonstrated the absorption of human milk IgG across the gut into the bloodstream.

The aim of this study was to determine the stability of recombinant palivizumabs (IgG, IgA and sIgA) and naturally-occurring human milk anti-RSV IgG and sIgA/IgA across gastric and intestinal phases of an *in vitro* model of infant digestion via a validated RSV F protein ELISA. The data from this work can be used to inform the design of recombinant antibodies for gastrointestinal pathogen prevention in the future.

## 2 Materials and methods

### 2.1 Sample collection

Four human milk samples were provided by four mothers through the Northwest Mother's Milk Bank (Portland, OR, USA). The informed consent procedure was managed by the Northwest Mother's Milk Bank. After obtaining informed consent from donors, milk samples were collected. All milk samples were from mothers who gave birth to infants at < 37 weeks gestational age and were collected from the first week of lactation. Samples were separated into 2-mL aliquots and kept at −80 °C.

### 2.2 *In vitro* digestion of individual mother’s milk

The *in vitro* infant gastrointestinal digestion method was described by (Nguyen, Bhandari, Cichero, & Prakash, 2015) with modifications. For gastric digestion, the four human milk samples (500 μL each) were supplemented with 100 μg/mL palivizumab IgG (Synagis®, MedImmune, Gaithersburg, MD, USA), palivizumab IgA and sIgA (Center for Global Infectious Disease Research, Seattle, WA, USA) (sequences shown in Supplementary Table 1 and the method for production is shown in Supplementary file 1) or not supplemented. Palivizumab IgA was prepared by combining palivizumab IgA1 and IgA2 (18 mg/mL) in a 3:2 (v/v) ratio as found in human breast milk (Chirico, Marzollo, Cortinovis, Fonte, & Gasparoni, 2008). Palivizumab sIgA was prepared by combining secretory component (5.5 mg/mL) with palivizumab IgA (18 mg/mL) in a 2:1 (v/v) ratio and incubating for 30 min at RT. A 250-μL aliquot of simulated gastric fluid (0.15 M NaCl, pH 4.0) and 0.3 mg pepsin (22.75 U/mg total protein, based on the assumption that human milk is 10 mg/mL protein) were added to the samples. Each sample was readjusted to pH 4.0 ± 0.03 with 1 M HCl. The samples were incubated at 37 °C with shaking at 300 rpm in an Eppendorf thermomixer® (Eppendorf, Hauppauge, NY, USA) for 0, 30 and 60 min. At each time point, a 90-μL aliquot was removed and stored at −20 °C. Simulated intestinal digestion was immediately carried out with the remaining 60 min-simulated gastric digestion sample as the starting material. The sample was adjusted to pH 8.0 ± 0.03 by adding 1 M NaOH. Bile salts (0.4 mg) (Sigma-Aldrich, St. Louis, MO, USA) were added to make a final sample concentration of 2 mM bile salts. Porcine pancreatin (0.1 mg) (8 × USP, Sigma-Aldrich) was added to provide 3.45 U of pancreatin/mg of total protein in the aliquot of the incubated sample. The sample was readjusted to pH 8.0 ± 0.03. The mixture was incubated at 37 °C with shaking at 300 rpm in the thermomixer for 0, 30, 60, 90 and 120 min. At each time point, a 90-μL aliquot was removed and stored at −20 °C.

### 2.3 RSV F protein-specific IgG, IgA and sIgA ELISA

The concentrations of palivizumab IgG, IgA and sIgA, and naturally-occurring RSV F protein-specific IgG and sIgA/IgA in *in vitro* digestion samples were quantified by ELISA as described by Lueangsakulthai, Sah, Scottoline, and Dallas (2020) with modifications. Nunc MaxiSorp 96-well plates (Thermo Scientific, Waltham, MA, USA) were coated at 4 °C overnight with 100 μL of 100 ng/mL human RSV pre-fusion F protein (Sino Biological, Wayne, PA, USA). In between steps, the plates were washed three times with 200 μL of 0.05% Tween-20 (Bio-Rad, Rockford, IL, USA) in phosphate-buffered saline using a Wellwash™ Versa microplate washer (Thermo Scientific). To prevent the nonspecific binding of the antibodies, plates were blocked with 150 μL of 1% bovine serum albumin (Thermo Scientific) in 0.05% Tween-20 (Bio-Rad) in phosphate-buffered saline for 1 h at RT. Samples were added (100 μL/well) in triplicate wells, at two preoptimized dilutions (800× and 1600× for palivizumab sIgA and IgA, 400× and 800× for palivizumab IgG, 10× and 20× for naturally-occurring sIgA/IgA and 2× and 4× for naturally- occurring IgG) and incubated for 1 h at RT. Palivizumab IgG, IgA and sIgA were used to make a standard curve on each plate (at a range of 0–1000 ng/mL). One hundred microliters of 0.16 μg/mL goat anti-human IgG conjugated with horseradish peroxidase (Bio-Rad) were added for RSV F protein-specific IgG detection. One hundred microliters of 0.5 μg/mL goat anti-human IgA with horseradish peroxidase (Bio-Rad) were added for RSV F protein-specific IgA and sIgA detection. Plates were incubated for 1 h at RT. The color was developed by adding 100 μL of 3,3′,5,5′-tetramethylbenzidine substrate (Thermo Scientific) for 5 min at RT. Fifty microliters of 2 N sulfuric acid were added to stop the reaction. Absorbance was measured at 450 nm with a spectrophotometer (SpectraMax M2, Molecular Devices). Data were interpreted using four-parameter logistic models to make the standard curve on each plate with R^2^ > 0.99 for goodness fit using Softmax® Pro 7.0 software.

### 2.4 RSV F protein-specific ELISA validation

Four human milk samples (500 μL each) were supplemented with 100 μg/mL palivizumab (IgG, IgA or sIgA). The RSV F protein-specific IgG, IgA and sIgA ELISA were validated for accuracy, precision, lower limit of quantification (LLOQ) and upper limit of quantification (ULOQ) as described by (Andreasson et al., 2015) with some modifications. The RSV F protein-specific IgG, IgA and sIgA ELISA were performed for all four human milk samples on the same day with two dilutions and in triplicate. The accuracy of the assays was measured via % error calculated as follows: % Error = (V_A_ − V_0_)/V_A_ × 100, where V_A_ represents the known value and V_o_ represents observed value. The precision of the assays was measured as the % coefficient of variation (CV), calculated from the following equation: % CV = Standard deviation (SD)/ Average × 100. The LLOQ was determined by identifying the lowest mean level of expected concentration above which the % CV < 20% and ULOQ by identifying the highest mean level of expected concentration below which the % CV < 20% for all samples. Based on this validation, we used only values that were above the LLOQ and below the ULOQ for sample quantification.

### 2.5 Statistical analysis

Paired *t*-tests were applied to compare the absolute concentrations and percentage stability of each antibody across the timepoints of simulated gastrointestinal digestion (GraphPad Prism software, version 8.2.1). One-way ANOVA with Tukey’s multiple comparison test was also applied to compare percentage stability after complete simulated gastrointestinal digestion among palivizumab IgG, IgA, sIgA and naturally-occurring RSV F protein-specific IgG and sIgA/IgA. Differences were designated significant at *p* < 0.05.

## 3 Results

### 3.1 RSV F protein-specific IgG, IgA and sIgA ELISA method validation

The RSV F protein-specific IgG, IgA and sIgA ELISAs were validated (Table 1). Four human milk samples spiked with either palivizumab IgG, IgA or sIgA were analyzed within a single day with three replicates and two dilutions for each sample. The RSV F protein-specific IgG, IgA and sIgA ELISAs were highly accurate, with 18.20, 7.02 and 14.59% error, respectively. The % CV of RSV F protein-specific IgG, IgA and sIgA ELISAs were 26.90, 11.61 and 7.75%, respectively which meets typical validation requirements for assay precision (CV < 30%) (Andreasson et al., 2015). The LLOQ of the RSV F protein-specific IgG, IgA and sIgA ELISAs were 1, 5 and 2.5 ng/mL, respectively. The ULOQ of the RSV F protein-specific IgG, IgA and sIgA ELISAs were 250, 250 and 100 ng/mL, respectively. The range of absorbance values for palivizumab IgG samples (diluted at 400× and 800×), palivizumab IgA and palivizumab sIgA samples (diluted at 800× and 1600×) were well within the linear range of each ELISA (between the LLOQ and ULOQ).

**Table 1:** Palivizumab RSV F protein-specific IgG, IgA and sIgA ELISA method validation.

Parameters	Palivizumab RSV F protein-specific IgG	Palivizumab RSV F protein-specific IgA	Palivizumab RSV F protein-specific sIgA
Error (%)	18.20	7.02	14.59
Precision (% CV)	26.90	11.61	7.75
LLOQ a	1 ng/mL	5 ng/mL	2.5 ng/mL
ULOQ b	250 ng/mL	250 ng/mL	100 ng/mL

a Lower limit of quantification.

b Upper limit of quantification.

### 3.2 Survival of palivizumab IgG, IgA and sIgA across simulated infant gastrointestinal digestion

**Fig. 1:**
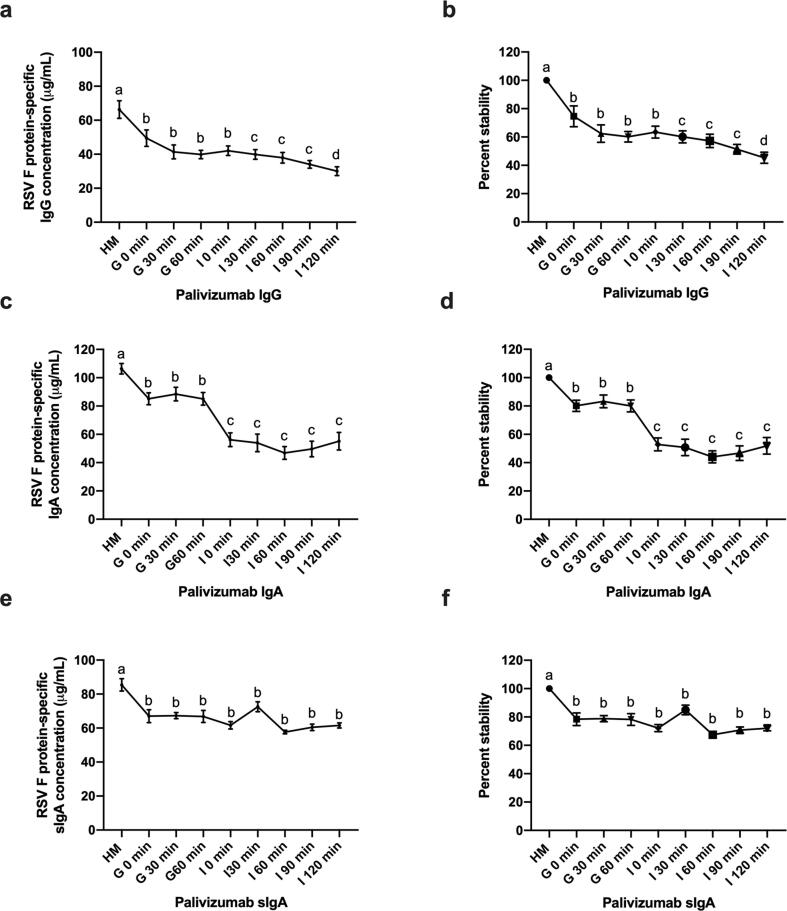
Concentration and percentage stability of palivizumab IgG, IgA and sIgA across simulated infant gastrointestinal digestion. (a) Average concentration of palivizumab IgG, (c) IgA and (e) sIgA from four human milk (HM) samples across gastric (G) and intestinal (I) digestion. (b) Percentage stability of palivizumab IgG, (d) IgA and (f) sIgA across gastrointestinal digestion. Values are mean ± SEM (*n* = 24), from four HM samples for each antibody, measured in triplicate at two dilutions and data were averaged. Letters *a*, *b*, *c* and *d* show statistically significant differences across gastrointestinal digestion time point (*p* < 0.01) using paired *t*-tests.

Concentrations of palivizumab in IgG, IgA and sIgA across simulated infant gastrointestinal digestion were determined by the RSV F protein-specific ELISA (average concentrations shown in Supplementary Table 2). Palivizumab IgG was degraded in both gastric and intestinal digestion (Fig. 1a and b). Palivizumab IgA was degraded in both gastric and intestinal digestion (Fig. 1c and d). Palivizumab sIgA decreased in gastric but was stable in intestinal digestion (Fig. 1e and f). Percentage stability of palivizumab IgG, IgA and sIgA all decreased across simulated gastrointestinal digestion (55%, 48% and 28% decrease, respectively). Palivizumab IgG and IgA were comparatively less stable than sIgA after complete simulated gastrointestinal digestion (*p* < 0.01).

### 3.3 Survival of naturally-occurring RSV F protein-specific IgG and sIgA/ IgA across simulated infant gastrointestinal digestion

**Fig. 2:**
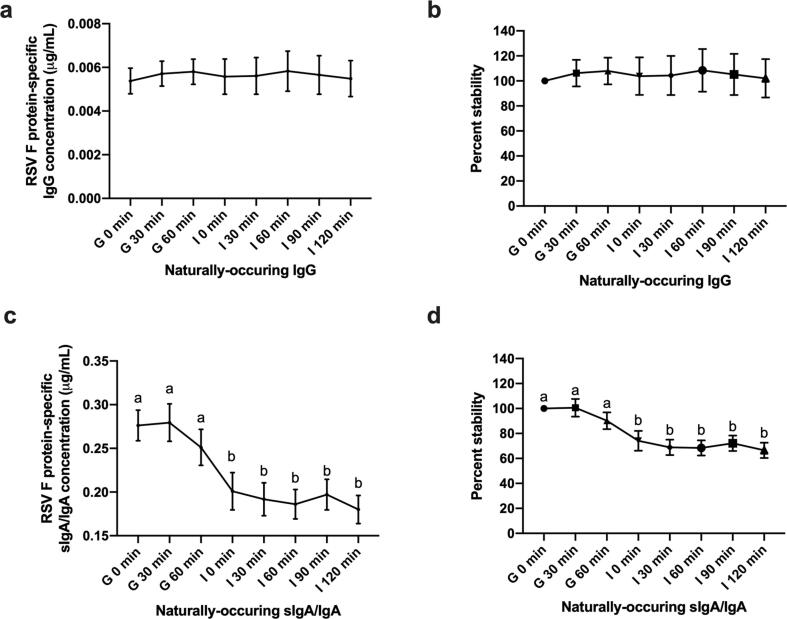
Concentration and percentage stability of naturally-occurring IgG and sIgA/IgA across simulated infant gastrointestinal digestion. (a) Average concentration of naturally-occurring IgG and (c) sIgA/IgA from four HM samples across gastric (G) and intestinal (I) digestion. (b) Percentage stability of naturally-occurring IgG and (d) sIgA/IgA across gastrointestinal digestion. Values are mean ± SEM (*n* = 24), from four HM samples for each antibody, measured in triplicate at two dilutions and data were averaged. Letters *a* and *b* show statistically significant differences across gastrointestinal digestion time point (*p* < 0.01) using paired *t*-tests.

**Fig. 3:**
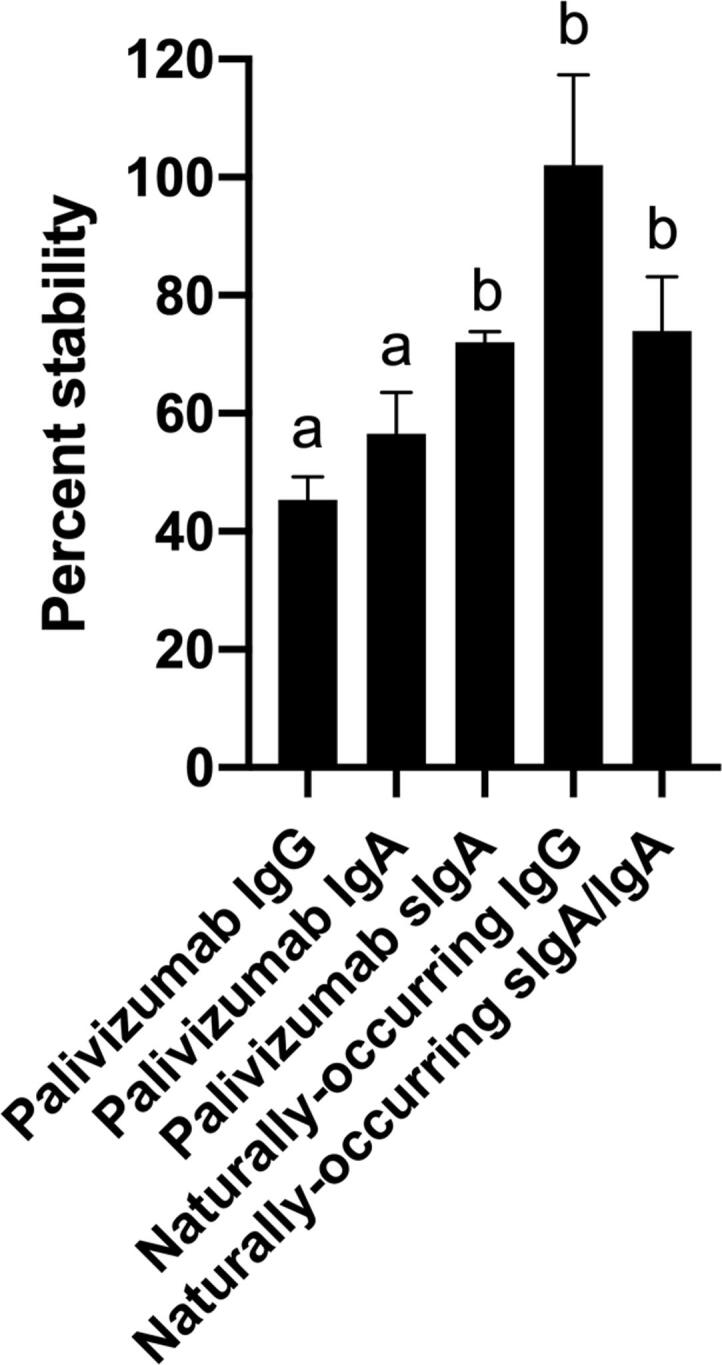
Percentage stability of palivizumab IgG, IgA and sIgA and naturally-occurring RSV F protein-specific IgG and sIgA/IgA after complete simulated gastrointestinal digestion. Values are mean ± SEM (*n* = 24), from four HM samples for each antibody, measured in triplicate at two dilutions and data were averaged. Letters *a* and *b* show statistically significant differences between samples (*p* < 0.01) using one-way ANOVA followed by Tukey’s multiple comparison test.

The average naturally-occurring RSV F protein-specific IgG concentration across four human milk samples was 0.005 μg/mL in milk, and the concentration did not decrease after *in vitro* infant gastric or intestinal digestion (Fig. 2a and b). Naturally-occurring RSV F protein-specific sIgA/IgA was presented at 0.276 μg/mL across four human milk samples, and this concentration did not decrease across time in simulated gastric digestion (30 min = 0.279, 60 min = 0.251 μg/mL) but was lower at 0, 30, 60, 90 and 120 min of simulated intestinal digestion (0.201, 0.191, 0.186, 0.197 and 0.180 μg/mL, respectively) (Fig. 2c and d). Overall, the percentage stability of naturally-occurring RSV F protein-specific sIgA/IgA decreased across simulated combined gastrointestinal digestion (33% decrease) (Fig. 2d), whereas IgG was stable across gastrointestinal digestion (Fig. 2b). Though RSV F protein-specific sIgA/IgA decreased across digestion, its overall percentage stability did not differ from naturally-occurring IgG. The overall stabilities of both naturally-occurring IgG and sIgA/IgA were higher than those of palivizumab IgG and IgA (Fig. 3). The average concentrations of naturally-occurring RSV F protein-specific sIgA/IgA in the four human milk samples was 32-fold higher than that of IgG (Supplementary Table 3).

## 4 Discussion

Enteric pathogen infection is a major problem among infants in developing countries. Infants are born with immature immune systems, including low numbers of immunoglobulin-secreting cells in the lamina propria at birth (Stoll et al., 1993). The production of immunoglobulin G (IgG) by infants is deficient, especially in premature neonates. Transplacental transfer of IgG from mother to infant only partially corrects this deficiency. Serum IgG decreases rapidly around three months of postnatal age (Lawrence & Pane, 2007). The development of vaccines against enteric pathogens represents a serious challenge because of the requirement to induce immunity that is effective in the gut. The induction of predominantly systemic immune responses by classical parenteral injection methods may not be optimal for inducing immunity to a pathogen colonizing the intestine (Dougan, Huett, & Clare, 2002). Although orally administered antibodies are susceptible to degradation by proteolytic enzymes in the gastrointestinal tract, several animal and human studies (Hatta et al., 1993, Hilpert et al., 1987, Shimizu et al., 1988) have shown that a fraction of orally administered polyclonal antibodies retain immunological activity. Therefore, orally administered antibodies may be useful for the treatment or prevention of local enteric pathogen infections (Reilly, Domingo, & Sandhu, 1997). In particular, recombinant monoclonal antibodies would be ideal to target a specific enteric pathogen. To be most effective, these recombinant monoclonal antibodies would need to survive across infant digestion, yet whether they survive remains unknown.

As a preliminary step for future work to testing the potential use of palivizumab in a future infant feeding study, we examined the *in vitro* digestion of palivizumab. Palivizumab is FDA-approved for use in neonates and infants via intramuscular injection, and thus may be a good candidate for *in vivo* studies of model specific immunoglobulin fate across infant digestion. By testing palivizumab IgG, IgA and sIgA, we examined how isotype affects stability across digestion. As human milk contains naturally-occurring anti-RSV antibodies, we also applied the same methodology to examine how the digestion stability of naturally-occurring antibodies compared with that of recombinant antibodies with similar antigen-specificity. This work is an essential step to inform optimal design of recombinant antibodies to increase the chances of success for using an oral antibody supplement to prevent enteric infections.

All three class formats of the palivizumab variable region were degraded across simulated gastrointestinal digestion. Though all palivizumab antibodies were degraded, palivizumab sIgA was more stable than IgA and IgG. The finding that the addition of secretory component increased the stability of IgA conforms with a previous finding that sIgA was stable in hostile environments, including the gut and mouth (Moldt, Saye-Francisco, Schultz, Burton, & Hessell, 2014). Though studies of the survival of recombinant antibodies in blood after injection exist (Liu, Manuilov, Chumsae, Babineau, & Tarcsa, 2011), we were unable to find previous studies examining *in vivo* gastrointestinal digestion of recombinant antibodies.

The naturally-occurring human milk RSV F protein-specific polyclonal IgG and sIgA/IgA were stable across the gastric phase of digestion. The findings of relatively high stability of naturally-occurring antibodies in the gastric phase of digestion confirms the results of our previous study (Demers-Mathieu et al., 2019) indicating that anti-influenza H1N1 hemagglutinin IgA and IgG were not digested in gastric contents of 20 premature infants fed mother’s milk. Anti-influenza H3N2 neuraminidase IgA and IgG were not digested in the stomach of infants fed mother’s milk at 8–9 days of postnatal age, but IgG was digested at 21–22 days of postnatal age. The present study results agree in that, for the most part, no digestion of naturally-occurring antibodies occurred in the infant stomach. Human milk RSV F protein-specific IgG was stable across complete simulated gastrointestinal digestion, but sIgA/IgA decreased during the intestinal phase of digestion. However, both palivizumab IgG and IgA were less stable across complete *in vitro* digestion than naturally-occurring RSV F protein-specific polyclonal sIgA/IgA. These results indicate that recombinant monoclonal IgG and IgA were less stable than milk polyclonal IgG and sIgA in simulated gastrointestinal digestion. The stability of naturally-occurring IgG in simulated infant digestion conforms with a previous study showing that orally supplemented bovine colostrum IgG at least partially survives digestion to the ileum even in adults (Roos et al., 1995). The lower stability of naturally-occurring sIgA/IgA compared with IgG conforms with a previous study (Eibl, Wolf, Fürnkranz, & Rosenkranz, 1988) of low birth weight infants orally-supplemented with naturally-occurring human serum-derived IgG and IgA, indicating the survival to the stool of IgG but not IgA. Taken together, these studies indicate that naturally-occurring IgG is resistant to infant digestion and that naturally-occurring sIgA/IgA is more stable than recombinant palivizumab IgG and IgA. This finding conforms with our previous study which indicated that naturally-occurring RSV F protein-specific polyclonal IgG and sIgA/IgA were more stable across *ex vivo* gastrointestinal digestion than palivizumab IgG, IgA and sIgA (Lueangsakulthai, Sah, Scottoline, & Dallas, 2020). Naturally-occurring antibodies may have greater resistance to digestion because of their differing structures compared with the structures of recombinant antibodies. However, it remains unclear how the naturally-occurring and recombinant antibodies differ in structure beyond their polyclonal vs. monoclonal nature. The glycosylation pattern of palivizumab IgG appears to be highly similar to that of naturally-occurring milk IgG (Goonatilleke et al., 2019, Hiatt et al., 2014, Huang et al., 2017).

RSV F protein-specific sIgA/IgA was more abundant than IgG in the four human milk samples tested. This finding aligns with those of a previous study from 454 breast milk samples that RSV F protein-specific IgA was more abundant than IgG (Mazur et al., 2019).

Our findings indicate that recombinant IgG and IgA may not be adequately resistant to digestion to optimally function to prevent infection in the infant gastrointestinal system without dosage compensation to account for loss due to proteolysis. Recombinant sIgA was more resistant to degradation. Naturally-occurring antibodies were more resistant to degradation than monoclonal antibodies.

## 5 Conclusions

To provide the best chances for success for an oral antibody supplementation approach with recombinant antibodies, recombinant sIgA may be the most stable antibody of choice. However, as naturally-occurring IgG appears to be more stable than naturally-occurring sIgA/IgA, it may be preferable to rationally engineer an IgG recombinant antibody that is more stable than the sIgA form. Such rational engineering may include examining and adopting the structural attributes of naturally-occurring antibodies that provide increased structural integrity in balance with retention of high activity. In their current forms, recombinant IgG and IgA still have potential to prevent infection but may need to be provided at a higher dose to compensate for digestive losses or be formulated or encapsulated to protect against degradation.

## 6 Ethics statement

This study was conducted according to the guidelines of the Declaration of Helsinki and the informed consent procedure was managed by the Northwest Mother's Milk Bank, Portland, USA. After obtaining informed consent from donors, milk samples were collected.

## CRediT authorship contribution statement

**Jiraporn Lueangsakulthai:** Conceptualization, Methodology, Investigation, Writing - original draft, Writing - review & editing. **Baidya Nath P. Sah:** Conceptualization, Writing - review & editing. **Brian P. Scottoline:** Conceptualization, Funding acquisition, Writing - review & editing. **David C. Dallas:** Conceptualization, Funding acquisition, Supervision, Writing - review & editing.

## Declaration of Competing Interest

The authors declare that they have no known competing financial interests or personal relationships that could have appeared to influence the work reported in this paper.
