# Supplementary material for: Survival of recombinant monoclonal and naturally-occurring human milk immunoglobulins A and G specific to respiratory syncytial virus F protein across simulated human infant gastrointestinal digestion
Source: J Funct Foods. 2020 Oct;73:104115. doi: 10.1016/j.jff.2020.104115 (PMC7573813; doi:10.1016/j.jff.2020.104115)
Supplement: Supplementary data 1 [file mmc1.docx]

**Supplementary file 1** Palivizumab IgA and sIgA construction design, expression and purification.

**Construct design**

Constructs encoding antibody heavy and light chains were built from gBlock DNA fragments (IDT) and assembled using NEBuilder HiFi DNA Assembly Master Mix (NEB). Namely, the kappa-acceptor vector was built using a DNA fragment encoding a leader sequence from murine variable kappa chain (ATGGGTGTGCCCACTCAGGTCCTGGGGTTGCTGCTGCTGTGGCTTACAGATGCCAGATGC), separated by a NotI restriction site from the human kappa-chain constant region (IGKC*01) in the pcDNA3.4 (Invitrogen) backbone plasmid. Similarly, two alpha-chain acceptor vectors were built using a DNA fragment encoding a leader sequence from a murine variable heavy chain(ATGGAATGGAGCTGGGTCTTTCTCTTCTTCCTGTCAGTAACTACAGGTGTCCACAGC), separated by a NotI restriction site from the human alpha-chain constant regions (IGHA1*01 and IGHA2*01, each resulting in its own acceptor vector) in the pcDNA3.4 (Invitrogen) backbone plasmid. Additionally, a gamma-chain acceptor vector was generated using a DNA fragment encoding a leader sequence from a murine variable heavy chain, separated by a NotI restriction site from the human gamma-chain constant region (IGHG1*02) in the pcDNA3.4 (Invitrogen) backbone plasmid. Acceptor vectors were linearized by NotI (NEB) digestion and used in assembly with their respective variable-gene segments encoding Palivizumab (Synagis®) (GenBank: KC283077, KC283078) using NEBuilder HiFi DNA Assembly Master Mix (NEB), resulting in Palivizumab.hIgG1, Palivizumab.hIgA1, Palivizumab.hIgA2, Palivizumab.hIgK plasmids. A gBlock encoding human J-chain (hIgJ) sequence (GenBank: XM011531926) was assembled with the pcDNA3.4 (Invitrogen) backbone plasmid using NEBuilder HiFi DNA Assembly Master Mix (NEB). Human polymeric immunoglobulin receptor (hPIGR) construct was assembled from a cDNA clone (Sino Biological, cat. HG10131-UT), which was used to extract the fragment encoding the hPIGR ectodomain (amino acids 19–638), and place it between DNA fragments encoding tissue plasminogen activator leader peptide (Wang et al., 2011) and a C-terminal 8xHis tag followed by an AviTag (Fairhead & Howarth, 2015) into the pcDNA3.4 backbone plasmid, resulting in the hPIGR.HisAvi plasmid.

### **Protein expression and purification**

Protein production was performed using transient transfection of HEK293F cells, as previously described (Carbonetti et al., 2017). Briefly, recombinant hPIGR was produced by high-density PEI transfection with the hPIGR.HisAvi construct. Following 5 days of culturing, conditioned medium was harvested by centrifugation, and supplemented by the addition of NaN_3_ (0.02% final concentration) and NaCl (+350 mM, final concentration). Protein was then purified by immobilized metal-affinity chromatography using HisPur Ni-NTA resin (Thermo Scientific) followed by gel-filtration over a HiLoad 16/600 Superdex 200 pg column (GE Healthcare). For antibody production, HEK293F cells were co-transfected as follows: hIgG1/hIgK plasmids were used at 0.5/0.5 ratio, whereas hIgA1/hIgK/hIgJ or hIgA2/hIgK/hIgJ plasmids were used at 0.25/0.25/0.5 ratio. Following 5 days of culturing, conditioned medium was harvested by centrifugation, supplemented by the addition of NaN_3_ (0.02% final concentration), and NaCl (+350 mM, final concentration). Protein was then captured using Pierce Protein A Plus (Thermo Scientific), for IgG purification, or Pierce Protein L Plus Agarose (Thermo Scientific), for dIgA purification, washed with HBS-E-hs buffer (10 mM HEPES, pH 7, 300 mM NaCl, 2 mM EDTA), and eluted in 0.1 mM glycine, pH 2.7 (fractions were immediately pH-neutralized using 1 M Na_2_HPO_3_). Protein-containing fractions were pooled and buffer-exchanged against HBS-E (10 mM HEPES, pH 7, 150 mM NaCl, 2 mM EDTA) by ultrafiltration.

**References**

Carbonetti, S., Oliver, B. G., Vigdorovich, V., Dambrauskas, N., Sack, B., Bergl, E., Kappe, S. H. I., & Sather, D. N. (2017). A method for the isolation and characterization of functional murine monoclonal antibodies by single B cell cloning. *Journal of Immunological Methods, 448*, 66-73.

Fairhead, M., & Howarth, M. (2015). Site-specific biotinylation of purified proteins using BirA. *Methods in molecular biology 1266*, 171-184.

Wang, J.-Y., Song, W.-T., Li, Y., Chen, W.-J., Yang, D., Zhong, G.-C., Zhou, H.-Z., Ren, C.-Y., Yu, H.-T., & Ling, H. (2011). Improved expression of secretory and trimeric proteins in mammalian cells via the introduction of a new trimer motif and a mutant of the tPA signal sequence. *Applied Microbiology and Biotechnology, 91* (3), 731-740.
