# Supplementary material for: Survival of recombinant monoclonal and naturally-occurring human milk immunoglobulins A and G specific to respiratory syncytial virus F protein across simulated human infant gastrointestinal digestion
Source: J Funct Foods. 2020 Oct;73:104115. doi: 10.1016/j.jff.2020.104115 (PMC7573813; doi:10.1016/j.jff.2020.104115)
Supplement: Supplementary data 2 [file mmc2.docx]

**Supplementary Table 1** The sequences of dimeric human IgA1, IgA2. Only the variable segment sequences are derived from palivizumab (Genbank: KC283077, KC283078). The sequences with variable-segment sequences were highlighted.

| Formats | Sequences |
| --- | --- |
| Palivizumab IgA1 | QVTLRESGPALVKPTQTLTLTCTFSGFSLSTSGMSVGWIRQPPGKALEWLADIWWDDKKDYNPSLKSRLTISKDTSKNQVVLKVTNMDPADTATYYCARSMITNWYFDVWGAGTTVTVSSASPTSPKVFPLSLCSTQPDGNVVIACLVQGFFPQEPLSVTWSESGQGVTARNFPPSQDASGDLYTTSSQLTLPATQCLAGKSVTCHVKHYTNPSQDVTVPCPVPSTPPTPSPSTPPTPSPSCCHPRLSLHRPALEDLLLGSEANLTCTLTGLRDASGVTFTWTPSSGKSAVQGPPERDLCGCYSVSSVLPGCAEPWNHGKTFTCTAAYPESKTPLTATLSKSGNTFRPEVHLLPPPSEELALNELVTLTCLARGFSPKDVLVRWLQGSQELPREKYLTWASRQEPSQGTTTFAVTSILRVAAEDWKKGDTFSCMVGHEALPLAFTQKTIDRLAGKPTHVNVSVVMAEVDGTCY |
| Palivizumab IgA2 | QVTLRESGPALVKPTQTLTLTCTFSGFSLSTSGMSVGWIRQPPGKALEWLADIWWDDKKDYNPSLKSRLTISKDTSKNQVVLKVTNMDPADTATYYCARSMITNWYFDVWGAGTTVTVSSASPTSPKVFPLSLDSTPQDGNVVVACLVQGFFPQEPLSVTWSESGQNVTARNFPPSQDASGDLYTTSSQLTLPATQCPDGKSVTCHVKHYTNPSQDVTVPCPVPPPPPCCHPRLSLHRPALEDLLLGSEANLTCTLTGLRDASGATFTWTPSSGKSAVQGPPERDLCGCYSVSSVLPGCAQPWNHGETFTCTAAHPELKTPLTANITKSGNTFRPEVHLLPPPSEELALNELVTLTCLARGFSPKDVLVRWLQGSQELPREKYLTWASRQEPSQGTTTFAVTSILRVAAEDWKKGDTFSCMVGHEALPLAFTQKTIDRLAGKPTHVNVSVVMAEVDGTCY |
