# Supplementary material for: Survival of recombinant monoclonal and naturally-occurring human milk immunoglobulins A and G specific to respiratory syncytial virus F protein across simulated human infant gastrointestinal digestion
Source: J Funct Foods. 2020 Oct;73:104115. doi: 10.1016/j.jff.2020.104115 (PMC7573813; doi:10.1016/j.jff.2020.104115)
Supplement: Supplementary data 3 [file mmc3.docx]

**Supplementary Table 2** Average concentrations of palivizumab RSV F-protein specific IgG, IgA and sIgA from four human milk samples.

| Samples | Average antibody concentrations in simulated gastric digestion (μg/mL) ^a^ | | | Average antibody concentrations in simulated intestinal digestion (μg/mL) ^a^ | | | | |
| --- | --- | --- | --- | --- | --- | --- | --- | --- |
|  | 0 min | 30 min | 60 min | 0 min | 30 min | 60 min | 90 min | 120 min |
| Palivizumab IgG | 49.46 | 41.38 | 39.85 | 42.07 | 39.85 | 37.93 | 34.05 | 30.04 |
| Palivizumab IgA | 85.16 | 88.49 | 85.09 | 56.20 | 53.92 | 46.86 | 49.65 | 55.17 |
| Palivizumab sIgA | 67.00 | 67.32 | 66.81 | 61.64 | 72.55 | 57.64 | 60.44 | 61.54 |

**^a^** Values are mean, *n* = 24.
