# Supplementary material for: Survival of recombinant monoclonal and naturally-occurring human milk immunoglobulins A and G specific to respiratory syncytial virus F protein across simulated human infant gastrointestinal digestion
Source: J Funct Foods. 2020 Oct;73:104115. doi: 10.1016/j.jff.2020.104115 (PMC7573813; doi:10.1016/j.jff.2020.104115)
Supplement: Supplementary data 4 [file mmc4.docx]

**Supplementary Table 3** Average concentrations of naturally-occurring RSV F-protein specific IgG and sIgA/IgA from four human milk samples.

| Samples | Average antibody concentrations in human milk samples (μg/mL) ^a^ |
| --- | --- |
| Naturally-occurring IgG | 0.008 ± 0.005 |
| Naturally-occurring sIgA/IgA | 0.258 ± 0.094 |

**^a^** Values are mean ± SD, *n* = 24.
